# The Role of ^68^ Ga PSMA Imaging in Evaluating Adrenal Lesions in Prostate Cancer Patients

**DOI:** 10.1055/s-0044-1786012

**Published:** 2024-04-23

**Authors:** Funda Üstün, Büşra Özdemir Günay, Fethi Emre Ustabaşıoğlu, Selçuk Korkmaz

**Affiliations:** 1 Department of Nuclear Medicine, Faculty of Medicine, Trakya University, Edirne, Türkiye; 2 Department of Radiology, Faculty of Medicine, Trakya University, Edirne, Türkiye; 3 Department of Biostatistics, Faculty of Medicine, Trakya University, Edirne, Türkiye

**Keywords:** adrenal gland metastases, ^68^ Ga-PSMA, prostate cancer, benign adrenal gland, survival

## Abstract

**Objectives** Gallium-68 prostate-specific membrane antigen ( ^68^ Ga-PSMA) imaging is valuable for staging because an accurate diagnosis, metastatic or nonmetastatic for prostate cancer patients, is required for deciding to treatment approaches and prognostic assessment. The aim of this study was primarily to distinguish between benign and metastatic adrenal gland lesions detected during ^68^ Ga-PSMA positron emission tomography (PET)/CT imaging, to evaluate the presence of factors predicting its development, and then to determine the life expectancy of patients with metastatic adrenal lesions.

**Materials and Methods** We performed a database search for PET/CT records generated from June 2016 to February 2021 for “adrenal gland” in report for patients who underwent ^68^ Ga-PSMA examination with prostate cancer patients.

**Results** Twenty-three patients (10 benign and 13 metastatic) were included in this study. The total prostate-specific antigen, adrenal gland size, adrenal gland density, and maximum standardized uptake (SUVmax) values are significantly different between groups ( *p* < 0.05). On receiver operating characteristic curve analysis, the SUVmax cutoff value > 6.8 provided both sensitivity and specificity of 100%. However, with 29 mm as the adrenal gland size cutoff and 21.2 as Hounsfield unit, the sensitivity and specificity were 56.2 and 92.3%, and 93.8 and 92.3%, respectively. The survival of the benign and metastatic groups was compared and a statistically significant difference was found ( *p* = 0.006). The presence of pelvic lymph nodes was statistically negatively affected the surveillance between the groups.

**Conclusion** The presence of atypical metastases such as adrenal gland is not insignificant in prostate cancer patients. Because of this degree of impact on patient management, accurate staging by imaging with ^68^ Ga-PSMA should be an integral part of prostate cancer management.

## Introduction

Prostate cancer represents the second most frequent cancer and the fifth leading cause of cancer death in men worldwide. 1 Differentiating nonmetastatic, oligometastatic, and multimetastatic diseases in prostate cancer has become critical in recent years.

The current European Association of Urology guidelines recommend at least one cross-sectional imaging modality (contrast-enhanced computed tomography [CT] or magnetic resonance imaging [MRI]) of the abdomen and the pelvis with radionuclide bone scintigraphy for locoregional and distant metastatic staging. 2 Overall, the most frequent sites for metastatic prostate cancer are bone (84%) and regional lymph nodes; however, dissemination to the lung, brain, and liver may also occur. 3 Distant metastasis, particularly visceral metastasis, is an important negative prognostic factor in prostate cancer, resulting in a significant increase in cancer-related mortality. So, these patients require aggressive treatment. 4 In the light of these developments, the conventional imaging might have limitations to evaluate prostate cancer. Therefore, additional imaging methods seem to be necessary even in the first stage.

Prostate-specific membrane antigen (PSMA) is a transmembrane glycoprotein that is overexpressed on prostate cancer cells. Its level of expression increases with increasing tumor aggressiveness. Gallium-68 ( ^68^ Ga) is obtained from a long-lived germanium-68/ ^68^ Ga generator. The 68-minute half-life of ^68^ Ga is appealing in which the patients are exposed to a relatively lower radiation dose. Imaging with ^68^ Ga-PSMA is based on the fact that it specifically binds to PSMA which is a cell surface protein, with significant overexpression in prostate cancer cells. 5 Although ^68^ Ga-PSMA is mostly used for secondary staging, it provides better accuracy in detecting lesions in primary staging, and thus significantly changed the paradigm for metastatic assessment despite the lack of guideline recommendations. 5 In a systematic review and meta-analysis study involving 37 articles and 4,790 patients, the pooled sensitivity and specificity per lesion and per patient were 75 and 99%, and 77 and 97%, respectively. 6

The adrenal glands are a common site for metastases, mainly because of their rich sinusoidal blood supply. Metastasis to the adrenal gland is most commonly caused by lung cancer, but it can also be caused by a variety of other primary malignancies, including renal cell carcinoma, malignant melanoma, breast, colon, and liver cancer. Although the number varies according to publications, adrenal gland metastasis is quite rare in prostate cancer (<1%). 4 7 8 However, based on some autopsy series and retrospective analysis, adrenal metastasis was found at significantly higher rates (∼20%). 9 10 11

When an adrenal mass is detected in the images, adrenal lesions could be divided into categories such as functioning or nonfunctioning masses, primary or metastatic, and benign or malignant. Differentiating benign from malignant adrenal disease is critical because an accurate diagnosis will inform management, which can include doing nothing, further investigation, or instituting definite local and/or systemic therapy. In CT, architectural features, such as size, shape, and homogeneity, the presence of intracellular lipid with Hounsfield units (HU) can be helpful in differential diagnosis. Like CT, MRI and nuclear imaging have also been used to distinguish between benign and malignant adrenal diseases. However, when the current literature is reviewed, there is no comprehensive study about distinguishing benign and malignant/metastatic adrenal lesions in patients with prostate cancer using ^68^ Ga-PSMA positron emission tomography (PET)/CT.

Our aim in this study was (1) to determine the distinction between benign and malignant adrenal gland lesions detected during ^68^ Ga-PSMA PET/CT imaging, (2) to determine the life expectancy of patients with metastatic adrenal lesions, and (3) to detect metastases accompanying adrenal gland metastases.

## Materials and Methods

### Patients

This retrospective study was approved by the local ethics committee (no.: TUTF-BAEK-2021/103).

We performed a database search for PET/CT records generated from June 2016 to February 2021 for “adrenal gland” in report for patients who underwent ^68^ Ga-PSMA PET/CT examination for the workup with prostate cancer patients. In this period, a total of 1,450 studies of ^68^ Ga-PSMA PET/CT were evaluated.

Inclusion criteria were determined as follows: all patients had a radiologic lesion in adrenals with or without ^68^ Ga-PSMA uptake. All lesions confirmed by a radiologist for second glance from PET/CT's CT component (F.E.U.).

Exclusion criteria were specified as the patients did not show any radiologic or pathologic adrenal lesions or ^68^ Ga-PSMA activity in adrenals. Patients were excluded from this study if clinical data were not complete or viewed with different radiopharmaceuticals such as fluorodeoxyglucose F18.

Demographic and clinic features, and pathologic characteristics were determined in each patient and recorded.

### PET/CT Images

^68^ Ga-labeled-conjugated PSMA-I&T was synthesized by a qualified staff using a fully automated Scintomics synthesis unit (Lindach, Fürstenfeldbruck, Germany). Quality control of the ^68^ Ga-PSMA-I&T radionuclide was performed by validated high-performance liquid chromatography and thin-layer chromatography according to the methods developed by others and described elsewhere.

Patients were not fasted and were allowed to take medications they used. They were well hydrated with 500 mL of oral water prior to the study. All patients underwent a single injection of ^68^ Ga-PSMA-I&T (1.8–2.2 MBq/kg [0.049–0.060 mCi/kg]). After administration of ^68^ Ga-PSMA-I&T, 20 mg of intravenous furosemide was injected to all patients, and they were provided emptying the bladder immediately before imaging.

After an uptake time of ∼60 minutes, image acquisitions were conducted in supine position (Discovery STE; GE Medical Systems, Milwaukee, Wisconsin, United States). All patients were examined using a three-dimensional mode with a 3-minute acquisition and six or seven bed positions without intravenous contrast injection. PET/CT images of lower extremities were added for all patients as per our institutional protocols. After decay and scatter correction, PET data were reconstructed iteratively with attenuation correction and reoriented in axial, sagittal, and coronal slices for three-dimensional reconstruction.

### Image Analysis

The lesions that were defined as “adrenal lesions” were re-evaluated for the nature and metabolic view of the lesions by an experienced radiologist (F.E.U.) and nuclear medicine expert (F.U.).

The existing CT and PET components of the study were reviewed at the workstations. The CT component of the study was used to measure the size, appearance, and lipid content of the adrenal gland. First of all, the lipid content of the adrenal mass was evaluated by noncontrast CT. Baseline evaluation of malignancy was made through this imaging. HU, calculated as less than or equal to 10 on noncontrast CT, is specific for lipid-rich lesions. This finding was evaluated in favor of adenoma. In addition, these adrenal lesions were evaluated according to some other radiological characteristic criteria such as appearance (integrity and invasiveness), heterogeneity, borders, and size. A radiologist (F.E.U.) is a physician experienced in this field, and using these listed parameters, he identified benign and malignant adrenal glands by “expert consensus.”

Adrenal gland lesions that radiologically approved by the radiologist were defined either when the ^68^ Ga-PSMA uptake had increased or no pathologic activity. ^68^ Ga-PSMA accumulation was analyzed semiquantitatively by calculating the maximum standardized uptake (SUVmax) value in the regions of interest (ROIs) placed over the suspected lesions.

To calculate SUVmax, manually defined ROIs were drawn on the attenuation-corrected emission image throughout axial planes in which a suspicious lesion could be delineated.

### Statistical Analysis

The Shapiro–Wilk's test was used to assess univariate normality. The Student's *t* -test was used to compare two independent groups. The Pearson's chi-square test or Fisher's exact test was performed to investigate the relationships between categorical variables. The Kaplan–Meier analysis was used to assess the potential risk factors. Receiver operating characteristic (ROC) curve analysis was performed to identify cutoff values. Numerical variables were summarized as mean and standard deviation, while categorical variables as frequency and percentage. A *p* -value of less than 0.05 was considered statistically significant. All statistical analyses were performed using IBM SPSS version 20.

## Results

### Patient Characteristics and Outcomes

**Fig. 1:**
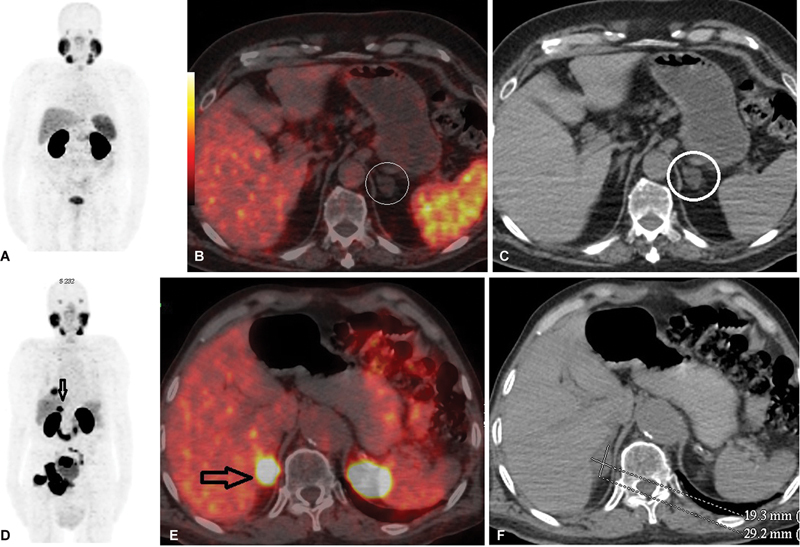
Comparison of PET/CT images showing benign and metastatic adrenal lesion patients. Panels ( **A–C** ) show a 69-year-old patient in the benign group with a Gleasson score of 5 + 4 and a PSA of 0.05. There is a benign lesion in the left adrenal gland with a long axis of 19 mm, −12 HU, SUVmax: 4.5 (white circle). In addition, the patient had perirectal lymph node (not shown) and bone metastasis in the sacrum. Panels ( **D–F** ) show an 83-year-old patient in the metastatic group with a total PSA of 0.7. Postimaging survival lasted 1 month. Long axis 29 mm, density 26.9 HU, SUVmax: 29.5 metastatic lesion (dark arrows) are observed in the right adrenal. The patient also has pelvic lymph node, multiple bone, and multiple lung metastases. ( **A, D** ) Maximum intensity projection images, ( **B, E** ) axial PET/CT images, and ( **C, F** ) axial CT images. CT, computed tomography; HU, Hounsfield unit; PET, positron emission tomography; PSA, prostate-specific antigen; SUVmax, maximum standardized uptake.

From June 2016 to February 2021, 1,450 patients were undergone ^68^ Ga-PSMA PET/CT imaging. When we searched their reports, 23 patients were tagged with a keyword “adrenal gland” (1.6%). Of the 23 patients included in this study, 10 of them were defined as benign group and 13 as metastatic group according to radiologic and metabolic appearances ( Fig. 1 ). Lesions were bilateral in 3 of 13 patients in the metastatic group and in 3 of the benign group.

Table 1 shows the characteristics of the patients. The mean age of men included in this study was 73 ± 6.9 and 70 ± 7.6 years in benign and metastatic groups, respectively.

**Table 1:** Principal characteristics of patients

	Benign group ( *n* = 10) (Mean ± SD)	Metastatic group ( *n* = 13) (Mean ± SD)	*p* -Value
Adrenal gland size (mm)	18.25 ± 9.9	24.06 ± 19.4	0.005
Adrenal gland density (HU)	5 ± 10	24 ± 18	0.006
Adrenal gland (SUVmax)	1.9 ± 1.4	13.4 ± 13.3	0.004
Gleasson (total)	8.5 ± 1.2	8.75 ± 0.9	ns
Total PSA (ng/mL)	7.3 ± 8.6	899.6 ± 1,446	0.000
Survival time (mo)	42 ± 5.6	12 ± 3.4	0.006

Abbreviations: HU, Hounsfield unit; ns, not significant; PSA, prostate-specific antigen, SD, standard deviation; SUVmax, maximum standardized uptake.

According to revised original Gleason score (grade group 1 [Gleason score ≤6], grade group 2 [Gleason score 3 + 4 = 7], grade group 3 [Gleason score 4 + 3 = 7], grade group 4 [Gleason score 8], and grade group 5 [Gleason scores 9–10]), 12 patients grade group at initial prostate biopsy, patients were classified as 3 (17.4%), 4 (17.4%), and 5 (52.2%). However, three patients (13%) could not be classified because their data were missing.

When the patient groups with metastatic and benign lesions are compared, there was no statistically significant difference in terms of age and Gleasson scores ( *p* > 0.05). However, total prostate-specific antigen (PSA), adrenal gland size, adrenal gland density, and SUVmax values were significantly different in statistical analysis ( *p* < 0.05).

The adrenal gland size, HU, and SUVmax were used to discriminate between benign and metastatic adrenal lesions. We found the optimal SUVmax cutoff to be 6.8 as determined by the ROC curve analysis, and the adrenal gland size, SUVmax, and HU for area under the curve were 0.69, 1, and 0.98, respectively. In SUVmax of 6.8 as the cutoff value, the sensitivity and specificity were 100 and100%, respectively. The adrenal gland size has low discrimination in distinguishing between metastasis and benign lesions (sensitivity and specificity, 56.2 and 92.3%, respectively). In HU of 21.2 as the cutoff value, the sensitivity and specificity were 93.8 and 92.3%, respectively.

### Survival and Prognostic Factors

Median follow-up time was 22 months (range 1–48 months) for all patients after the diagnosis of the adrenal lesions.

**Fig. 2:**
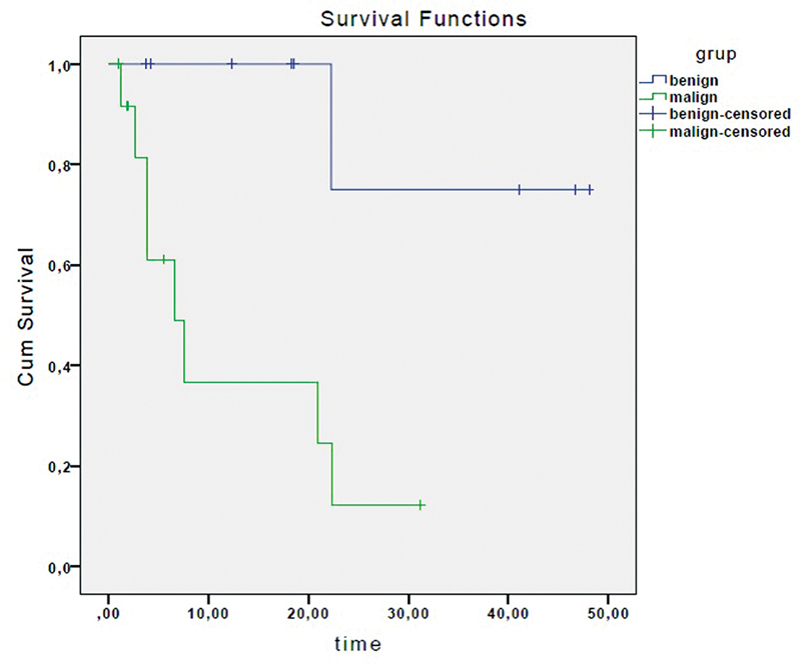
Overall survival curve of 23 patients with adrenal lesion by Kaplan–Meier analysis.

The survival of the benign and metastatic groups was compared and a statistically significant difference was found ( *p* = 0.006) ( Table 1 and Fig. 2 ).

Additionally, we evaluated the parameters affecting surveillance in this study population ( Table 2 ). It was observed that the presence of pelvic lymph nodes, which was the only factor statistically significant, negatively affected the surveillance in this benign and metastatic patient groups with a total of 23 prostate cancer patients ( *p* < 0.05). While patients without lymph node metastasis had a life expectancy of 39 ± 5.9 months, those with multiple lymph node metastases had a life expectancy of 18 ± 12.8 months. Additionally, although not statistically significant, other parameters also cause the considerable decline in the survey. These factors that affect survival also predict metastatic lesions. However, a statistically significant value could not be reached due to the small number of cases.

**Table 2:** Analysis of prognostic factors influencing the survey

Characteristics at scan time	Mean (95% CI)	*p* -Value
Pelvic lymph node metastasis		
No	39.31 (27.79–50.83)	0.011
< 5	14.09 (5.12–23.06)	
Multiple	18.4 (0.00–43.55)	
Bone metastases		
No	30.34 (13.22–47.46)	0.422
< 5	28.27 (12.64–43.90)	
Multiple	14.30 (9.76–18.84)	
Abdominal lymph node metastasis		
No	30.74 (19.37–42.10)	0.097
Yes	15.53 (7.83–23.23)	
Thoracic lymph node metastasis		
No	26.74 (14.74–38.74)	0.405
< 5	19 (17.93–20.06)	
Multiple	9.66 (0.49–18.83)	
Head and neck lymph node metastasis		
No	27.76 (18.87–36.65)	0.055
Yes	4.54 (1.95–7.13)	
Presence of lymph node except for loco regional area		
No	27.76 (13.28–42.23)	0.427
Yes	18.81 (12.10–25.52)	
Organ metastasis		
No	19.16 (7.81–30.52)	0.073
Yes	25.25 (20.06–43.99)	

Abbreviation: CI, confidence interval.

**Fig. 3:**
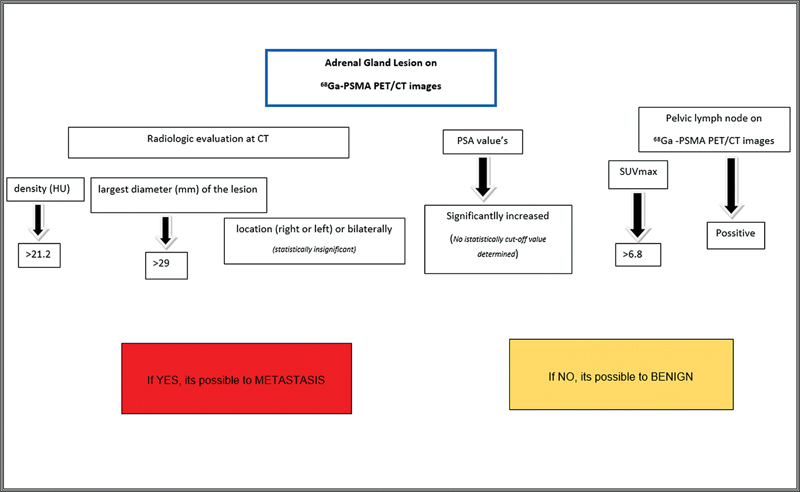
Based on this study, the differential diagnosis of lesions in the presence of adrenal gland in ^68^ Ga-PSMA PET/CT in patients with prostate cancer. CT, computed tomography; ^68^ Ga-PSMA, gallium-68 prostate-specific membrane antigen; HU, Hounsfield unit; PET, positron emission tomography PSA, prostate-specific antigen; SUVmax, maximum standardized uptake.

In the light of these data, we prepared a figure for the differential diagnosis of lesions in the adrenal gland at ^68^ Ga-PSMA ( Fig. 3 ).

## Discussion

The quantity or location of metastases in a cancer patient is a prognostic factor. 4 It has been shown in studies that the presence of visceral metastasis (e.g., liver metastasis, median 10 months) has a significant negative effect on surveillance compared with the presence of only lymph node (median 26 months) or only bone metastasis (median 19 months) in patients with prostate cancer. 13 Thus, they are less likely to respond to traditional treatment modalities and they might warrant more aggressive approaches. However, no data could be found on the effect of adrenal gland metastasis, which is a visceral organ, on surveillance in prostate cancer, when the literature is reviewed.

According to our study, we reached that adrenal involvement associated with more severe clinical manifestations. The survival rates significantly decreased according to the presence or absence of adrenal gland metastases. Therefore, it is very useful to evaluate patients with whole body imaging with ^68^ Ga-PSMA, which is a metabolic agent.

The standard imaging for prostate cancer includes CT and whole body technetium bone scans. Such imaging methods limited by poor sensitivity—especially in oligometastatic disease and low PSA levels. These limitations are particularly important at a lower and earlier stage of the disease and increased PSA levels. This situation encouraged the development of new imaging models in recent years. ^68^ Ga-PSMA PET also significantly influenced clinical decision-making and changed it in ∼50% of the patients. 14 ^68^ Ga-PSMA PET showed excellent sensitivity and specificity for localized or distant metastasis, for the treatment response or in case of recurrence. 6 In addition, with this study, it was found that there is a strong relationship between increased PSA levels and metastatic disease detection in ^68^ Ga-PSMA. According to this comprehensive meta-analysis, the rate of positive results reaches 97% when the PSA value is above 2 to below 0.2 compared with 33%. 6

In prostate cancer patient, although the rate of metastasis in the adrenal gland is so rare, adrenal masses identified during the staging or for re-evaluation are often assumed to be metastatic lesions. However, when an adrenal tumor is found in a cancer patient, this may indicate a process unrelated to the index malignancy. This misdiagnosis may result delayed or erroneous. For all these reasons, it is important to determine which patient had primary adrenal malignancy and which one had metastasis.

Architectural features such as size, shape, and homogeneity can be helpful in suggesting a diagnosis and distinguishing between benign and malignant. According to our study, SUVmax value of 6.8, HU value of 21.2, and adrenal gland size above 29 mm can be used as cutoff values in the differentiation of metastasis and benign adrenal glands. The combination of CT with or without contrast further improves the accuracy of ^68^ Ga-PSMA PET.

When the literature is reviewed, there are very different rates of adrenal gland metastasis in prostate cancer (0–23%). 3 7 9 10 11 15 16 There may be more than one reason why the results of the studies are so different: (1) the retrospective nature of the studies, (2) the different patient groups, (3) the use of autopsy data or patient's data from file records, (4) the increase in current treatment modalities resulting in much more patient to follow-up in advanced stages, (5) keeping data records smoother over the years, (6) advances in technology, and (7) overlooking adrenal gland lesions with other methods compared with ^68^ Ga-PSMA, which is a whole body imaging method.

In our study, the frequency of adrenal metastasis seems to be higher than previous studies. When evaluated together with the reasons listed earlier, this may be due to the above-mentioned reasons. These lesions had reportedly been missed with conventional imaging methods because local primary and salvage surgery and radiation therapy do not cover this area as a standard. Today, ^68^ Ga-PSMA imaging is used more and more frequently. A significant number of ^68^ Ga-PSMA are performed in our center (∼ 12–15/week). Our center is very experienced in this regard. At the same time, advanced stage patients are more common due to developing treatment modalities. Therefore, it is thought that adrenal metastasis rates are higher because advanced stage patients tend to screen more for PSMA. Considering all this, it has been concluded that the presence of adrenal metastasis is substantially important today, and adrenal gland metastasis rates will change toward an increase as a result of advances in imaging methods.

The adrenal gland is a retroperitoneal organ located in the suprarenal area bilaterally, with medial and lateral limbs looking like an inverted Y, V, or L in shape. Bazhenova et al 17 with a mathematical model called the “Newton” model simulated how a tumor cell moved from the primary site to the metastatic sites. In this article, while adrenal gland metastasis is originated from lung cancer, the mechanisms of metastasis to adrenal gland are pretty much same in other cancers. In prostate cancer, adrenal gland is defined as a sixth common site of metastasis. There are different hypotheses about metastasis in the adrenal gland, which is an atypical area in prostate cancer. The development of adrenal metastases can be thought to be related to the rich sinusoidal blood supply. In this condition, the patient should have adrenal metastasis without any other area. However, in our patient group, no single adrenal metastasis was observed without systemic spread of disease. All of these patients have lymph node and/or another organ metastasis. On the other hand, organ metastases with direct lymph drainage of primary tumors have been explained. In addition, according to the mechanical hypothesis, it is predicted that the tumor disseminated to the lymphatic system or body cavity first, then spread over a long distance through the venous system. The only factor predicting the presence of adrenal involvement in our metastatic patient group was the presence of pelvic lymph node metastasis. Other findings were not statistically significant. Therefore, lymphatic spread can be considered primarily as a mechanism of metastasis in the adrenal gland in prostate cancer. On the other hand, it has been suggested that aggressive histopathological diagnosis may lead to atypical metastasis areas such as the adrenal gland. However, there was no pathological difference in our cases between the aggressive or indolent histopathology. Therefore, it would be appropriate to conduct a prospective study especially patients with aggressive histopathology.

However, in adrenal gland metastasis, as well as in prostate primary tumors that metastasize to bone tissues, it is difficult to explain in terms of drainage patterns and suggests the presence of additional factors such as tissue compatibility (along the lines of the seed and soil hypothesis) and/or homing mechanisms (in line with more recent molecular findings). 3

Despite advances in medical treatment, we cannot explain the mechanisms of metastasis or progression patterns in all diseases. Further studies that investigating the biologic mechanisms underlying lung, liver, bone, and nodal metastatic spread are imperative. The presence of metastasis in the adrenal gland could not be explained by a single theory and suggested that there are more patterns of metastasis than factors involving blood flow and/or lymphatic drainage.

There are several limitations to our study. This study was retrospective in nature. The histopathological confirmation was not performed as the patients exhibited extensive disease and advanced stage. Since our imaging center is in the reference position, the clinical follow-up of some patients is in external centers. Therefore, some data of the patients are missing, such as the Gleasson score.

## Conclusion

In recent years, proper evaluation efforts have resulted in a huge increase in the imaging of prostate cancer patients, resulting in a larger pool of patients to investigate the disease's patterns and unusual areas of dissemination. However, less emphasis has been paid to documenting the imaging and clinical aspects of less common sites of prostate cancer metastases, such as the adrenal gland. ^68^ Ga-PSMA in prostate cancer is increasingly used to detect disease burden and metastasis because the results of ^68^ Ga-PSMA affect clinical practice and treatment management in about half of the patients. The presence of concomitant adrenal metastases affects on surveillance poorly according to the state of nonmetastasis in advanced stage disease. The progress in imaging techniques has led to an increasing diagnosis of adrenal metastases which were identified during tumor staging and follow-up examinations. PET has an excellent sensitivity and specificity for distinguishing benign from malignant adrenal lesions, and it seems like the best imaging technique for the evaluation and detection of adrenal masses in prostate cancer.
